# Food Insecurity in Greece and across the Globe: A Narrative Literature Review

**DOI:** 10.3390/foods13101579

**Published:** 2024-05-18

**Authors:** Emmanouil Alexandros Fotakis, Ioanna Kontele, Milia Tzoutzou, Maria G. Grammatikopoulou, Eirini Arvanitaki, Theodoros N. Sergentanis, Konstantinos Kotrokois, Eleni Kornarou, Tonia Vassilakou

**Affiliations:** 1 Department of Public Health Policy, School of Public Health, University of West Attica, 196 Alexandras Avenue, 11521 Athens, Greece; mdy20044@uniwa.gr (E.A.F.); ikontele@uniwa.gr (I.K.); earvanitaki@uniwa.gr (E.A.); tsergentanis@uniwa.gr (T.N.S.); kkotrokois@uniwa.gr (K.K.); ekornarou@uniwa.gr (E.K.); 2 Department of Nutrition and Dietetics, Hellenic Mediterranean University, 72300 Sitia, Greece; miltzu@hmu.gr; 3 Department of Rheumatology and Clinical Immunology, Faculty of Medicine, School of Health Sciences, University of Thessaly, Biopolis, 41110 Larissa, Greece; 4 Open Elderly Care Center, Municipality of Keratsini-Drapetsona, 18756 Athens, Greece

**Keywords:** insecurity, food, unemployment, poverty, health, hunger, pandemic, climate

## Abstract

Food insecurity comprises a major global public health threat, as its effects are detrimental to the mental, physical, and social aspects of the health and well-being of those experiencing it. We performed a narrative literature review on the magnitude of global food insecurity with a special emphasis on Greece and analyzed the major factors driving food insecurity, taking into consideration also the effect of the COVID-19 pandemic. An electronic search of international literature was conducted in three databases. More than 900 million people worldwide experience severe food insecurity, with future projections showing increasing trends. Within Europe, Eastern and Southern European countries display the highest food insecurity prevalence rates, with Greece reporting a prevalence of moderate or severe food insecurity ranging between 6.6% and 8% for the period 2019–2022. Climate change, war, armed conflicts and economic crises are major underlying drivers of food insecurity. Amidst these drivers, the COVID-19 pandemic had a profound impact on food insecurity levels around the globe, through halting economic growth, disrupting food supply chains and increasing unemployment and poverty. Tackling food insecurity through addressing its key drivers is essential to any progress towards succeeding the Sustainable Development Goal of “Zero Hunger”.

## 1. Introduction

While we are racing through the fourth industrial revolution of ground-breaking technological and scientific advancements, food insecurity remains an unresolved issue for humankind with profound impact on our societies, health and well-being [1].

Indicatively, according to the Food and Agricultural Organization (FAO) in 2020, one in three people across the globe did not have access to adequate food [1]. Food insecurity refers to (a) the uncertain or limited availability of nutritionally adequate and safe foods required for normal human growth and a healthy life and (b) the uncertain or limited ability to access such foods in socially acceptable ways (i.e., not live off charity-based food redistributions). In its broader sense, food insecurity also encompasses the pillars of food use or misuse (i.e., households and individuals must be able to utilize the food in a way that has a positive nutritional impact) [2,3].

Overarching the conditions of malnutrition, undernutrition, undernourishment, hidden hunger (i.e., micronutrient deficiency), hunger and starvation in multifarious contexts (e.g., high/borderline food security settings expressed as chronic, acute or transitory food insecurity) [4], food insecurity comprises a major global public health problem [5], which may affect health in multiple ways, including all three aspects of mental, physical and social well-being. Moreover, food insecurity may have detrimental effects on any life stage, whether in a cyclical continuum form (i.e., from pregnancy to infancy and childhood) or by developmental and life stage per se [6].

Notably, food insecurity, especially during childhood, is linked to increased morbidity and mortality [7,8]. It is estimated that nearly 45% of global child mortality is associated with malnutrition [9], while nearly 66 million children go to school hungry on a daily basis [10]. Amongst an array of detrimental effects in children, food insecurity is associated with suboptimal physical child development, such as stunting, mediated by inadequate food intake, but also increased susceptibility to infections and development of chronic diseases later in life (e.g., cardiovascular disease, obstructive pulmonary disease, cancers, asthma and autoimmune disease, and depression) [8,11]. Furthermore, food insecurity during childhood is associated with reduced learning and productivity, delayed speech development, behavioral problems, and poor social relationships [8]. In addition, childhood hunger is considered a risk factor both for depression and suicidal tendencies in adolescence and young adulthood [12,13]. 

An additional health concern is the association of food insecurity with obesity among both children and adults [1,14]. While food insecurity is not the generative cause of obesity, the intake of non-nutritious, energy-dense, low-cost foods in conjunction with food insecurity associated stress and downstream physiological adaptations (such as low birthweight and stunting in children) are associated with the occurrence of obesity later in life [14].

Food insecurity largely stems from poverty and economic inequalities at the individual, community and country level [15]. Within this causal context, identifying and analyzing key systemic macro-factors determining and co-driving food insecurity trends and their associated burden in our societies is key to informing and strengthening food security state policies and social actions. Moreover, analyzing the magnitude of food insecurity through a country case-study approach whilst also maintaining a global outlook may raise awareness and support multi-level evidence-based decision making [16].

Taking into consideration the complexity and immediate need for addressing the public health problem of food insecurity and from an advocacy standpoint of the WHO framework ‘’leave no one behind’’, alongside the 2030 interconnected sustainable development goals, ‘’Zero Hunger’’, “Zero Poverty”, “Reduce Inequalities” and ‘’Good Health and Well-being’’ [17], the aims of the current review are to (i) summarize the global magnitude of food insecurity with a special emphasis on Greece, as a developed European country experiencing a profound financial crisis in recent years further exacerbated by the COVID-19 pandemic, and (ii) analyze the major macro-factors driving food insecurity taking into consideration also the effect of the COVID-19 pandemic, especially during the pandemic’s acute emergency phase. 

## 2. Materials and Methods

### 2.1. Search Strategy and Information Sources

For the aims of this narrative review, an electronic search of international literature was conducted in three different databases (PubMeD/MEDLINE^®^ (US National Library of Medicine, Bethesda, MD, USA), Scopus (Elsevier, Amsterdam, The Netherlands) and Google Scholar (Google, Mountain View, CA, USA) from December 2021 until November 2023. The following keywords and Boolean operators were used in our search: (“food insecurity” OR “food security” OR “food deprivation” OR “malnutrition” OR “undernutrition” OR “undernourishment” OR “micronutrient deficiency” OR “overweight” OR “food waste” OR “hunger” OR “hidden hunger”) AND (“COVID-19 Pandemic” OR “financial crisis” OR “economic crisis” OR “war” OR “armed conflict” OR “climate change” OR “climate/Climatic crisis” OR “environmental degradation”), with or without defining geographic areas of interest (at continent or country level). 

In addition, we screened the websites of the World Health Organization (WHO), Food and Agriculture Organization (FAO), World Food Program, UNICEF, Greek Ministry of Health, Hellenic Statistical Authority and other organizations, institutes and institutions for any information referring to food insecurity between 2005 and 2023. Finally, data aggregator and visualization platforms obtaining primary data from official sources were also screened for relevant information [18].

### 2.2. Eligibility of Studies, Measures of Food Insecurity and Food Insecurity Categorization Levels

All types of observational studies, as well as reviews, meta-analyses and reports from Greek and international official authorities, were included. Only articles published in English were examined. Articles that had no full text available were excluded. No restriction was applied regarding the year of publication. In addition, no restriction was applied on the methods, metrics and indicators used for measuring food insecurity in the individual studies (i.e., Food Insecurity Experience Scale (FIES) survey module, which refers to household access to adequate food [19]; the Household Food Insecurity Access Scale (HFIAS) [20]; the Household Dietary Diversity Scale (HDDS) [21]; the Household Hunger Scale (HHS) [22]; the Coping Strategies Index (CSI) [23]; the Global Hunger Index [24]; and a series of other proxy measures). Finally, we retained the categorization levels of food insecurity (i.e., non-severe, mild, moderate, severe) as described in the original studies. Specifically, several indexes/scales such as HFIAS and HHS have commonly-used cut-offs determining food insecurity levels, although different assumptions and methods underlie each measure’s cut-offs. On the other hand, universal categorization thresholds are not available for other indexes/scales (e.g., CSI). The different indicators used for measuring food insecurity per study are described in Table 1 and Table 2.

## 3. Results

### 3.1. Magnitude of Food Insecurity

#### 3.1.1. The World at a Glance

Available studies point towards an increasing trend in food insecurity. From 777 million people affected by hunger globally in 2015, the number of people affected rose above 820 million in 2019 [2,24]. Latest estimates show that in 2022 approximately 900 million people experienced severe food insecurity (i.e., struggled to meet their energy needs), corresponding to 11.3% of the world’s population [25]. The sheer magnitude of the problem skyrockets when considering hidden hunger, which is estimated to affect approximately two billion people across the world (mostly children and pregnant women in developing and low-income countries) [31]. 

Food insecurity comprises a hallmark of health inequalities both between and within countries and is largely interconnected to the social determinants of health [32]. It affects different socioeconomic groups in diverse forms and levels of gravity, dependent also on the territorial context/geographical macro-area of residence [2]. 

The majority of the world’s undernourished persons (more than 400 million in 2022) live in Asia, whereas more than 280 million live in Africa which displays the highest increasing rate of undernourishment [18,23,25]. The remaining are split amongst Europe, Australia and the Americas. As a share of the population, severe food insecurity is highest in Sub-Saharan Africa, where almost 33% of the population are defined as severely insecure [16,18], whilst as of 2019 the projected mid-ranges of the prevalence of undernourishment exceeds 25% in Eastern and Central Africa [25]. Moderate or mild food insecurity (i.e., affecting those who worry or struggle about accessing a healthy, nutritious diet), although highest in South Asia and African countries, poses an important public health issue across the globe, including developed and high–income countries [18,26]. Regarding food crises (i.e., situations in which food quantity and/or quality drastically decrease within a short period of time), Yemen, the Democratic Republic of the Congo, Afghanistan, Venezuela and Ethiopia were the most severely affected countries in 2019 with a total of 60.1 million people affected by the crisis turmoil [1]. 

Overall, women appear to have a higher probability of being food insecure compared to men [27], whilst girls and adolescent females are more likely to report being food insecure than boys [2,33]. Particularly vulnerable are mothers with young children and pregnant women [3]. As regards children, the most vulnerable population group, in 2019 approximately 149 million children below the age of 5 years were stunted, nearly 50 million were wasted or subject to acute undernutrition, 340 million experienced systematic micronutrient deficiencies and 38.2 million were overweight or obese [28]. 

#### 3.1.2. Europe and Greece

Europe is characterized by satisfactory levels of food security, compared to other continents. Specifically, in 2010, food insecurity prevalence in Europe was recorded at 2.7%; i.e., a considerably lower rate compared to other regions of the world, although higher than what was expected for the region based on previous trends [2,34]. Nonetheless, the risk and burden of food insecurity is not homogeneous amongst European Member States [29]. Recent data generated via the FAO’s FIES shows that the prevalence rates of food insecurity in European countries range from 3.1% to over 20% [30], peaking in Eastern and Southern European countries [34]. 

With the onset of the economic crisis in 2008, food insecurity escalated in Greece [29]. In 2015, more than 1.4 million people were estimated to have experienced food insecurity, corresponding to 12.9% of the country’s population [35]. Moderate/severe food insecurity prevalence appears slightly higher during the period 2014–2018, estimated at 14.5% [36]. In 2019, the Hellenic Statistical Authority reported that 8% of the Greek population experienced moderate or severe food insecurity, with1.5% of the population experiencing severe food insecurity [37]. Similar prevalence rates were recorded in 2022 when 6.6% of the population experienced moderate or severe food insecurity and 1.5% severe food insecurity [37]. 

According to the results of the Hellenic Statistical Authority’s Income and Living Conditions Survey (SILC) in 2020, 13.2% of the population worried about not having enough food to cover their needs, 12.8% were not able to sustain a healthy and nutritious diet, 14.1% of the population consumed only certain food groups, 6.2% were forced to skip a meal, 6.6% consumed less food than what they believed was necessary for their needs, 2.7% of the households experienced low food adequacy, 3.0% of the population was hungry but did not eat, and 2.2% did not consume foods during a whole day [38].

Regarding the burden of food insecurity in specific population groups, Gatton and Gallegos [36] found that, during 2014–2018, Greece reported the highest prevalence of moderate/severe food insecurity (12.2%) in those aged 65 years and above among 34 high income countries. Notably, a cross-sectional study conducted in Northern Greece in 2017 focusing on older adults revealed that 69% of the study population experienced some degree of food insecurity, while it found a positive association between food insecurity and a lower educational level, reduced monthly income and low adherence to the Mediterranean Diet [39]. Moreover, in a second study from Greece conducted in 2019, the prevalence of elder participants’ food insecurity reached 50.4%, with men and older adults malnourished or at risk for malnutrition displaying higher odds of food insecurity [40]. Additionally, another study conducted in Greece in 2019 indicated that, nearly a decade following the onset of the economic crisis, a notable and concerning percentage of adult participants in a food assistance initiative still suffered from protein and energy deficiencies [41].

University students in Greece, also appear to be affected by a certain level of food insecurity. The study conducted by Theodoridis et al. (2018) on a non-probability sample revealed a significant proportion of students (45.3%) experiencing severe food insecurity, while increased food insecurity was inversely correlated to students adhering to the Mediterranean Diet [42]. 

Existing evidence indicates that the economic crisis in Greece had a serious impact on food security for the most vulnerable population groups, especially children and adolescents. During the 2012–2013 and 2014–2015 school years, 56.3% and 45% of children’s households residing in the poorer socioeconomic areas of Greece experienced high levels of food insecurity [43,44].

Furthermore, a study examining diet quality and food insecurity in pairs of mothers and their children in Greece in 2017 reported that more than 1/4 (26.3%) of the pairs reported some degree of food insecurity, with a greater prevalence (64.7%) within single-mother families [45], while a pilot study on refugee children living in two reception centers in Greece revealed that 13.0% of the participants had at least one form of malnutrition, 7.8% were underweight and 7.3% were affected by stunting [46].

The main findings regarding the magnitude of food insecurity globally, in Europe and in Greece are summarized in Table 1, Table 2 and Table 3.

### 3.2. Macro-Factors Driving Food Insecurity

**Figure 1:**
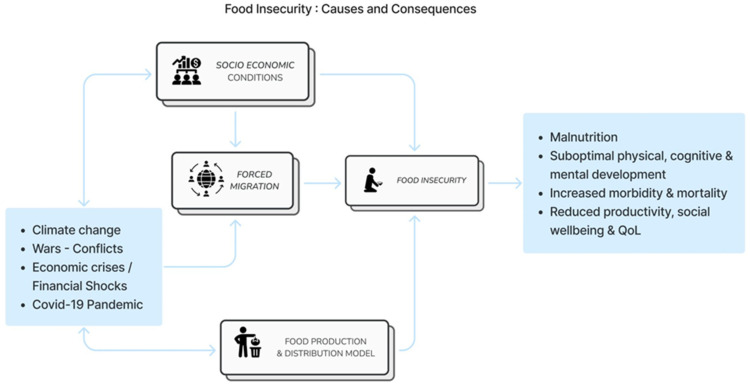
Root Causes and Health Consequences of Food Insecurity.

The root causes of food insecurity and their consequences on health are summarised in Figure 1.

#### 3.2.1. Climate Change

Climate change and climate extremes (i.e., droughts, floods, heatwaves, and other phenomena) are currently undermining food availability, access, utilization and stability in many ways resulting in a downward spiral of increasing food insecurity and food crises [1]. 

Hunger is substantially worse in countries and regions with agricultural systems sensitive to droughts, rainfalls, and temperature changes and where the population’s livelihood is tied to agriculture production and agricultural product consumption [1]. Notably, between 2008 and 2018, climate induced extreme weather events and associated disasters in low- and middle-income countries caused an estimated crop and livestock production loss of 6.9 trillion kilocalories per year, which corresponds to the yearly energy intake of 7 million adults [47].

As global warming increases, the risks to food security become more serious and complex. Regarding agricultural production, the increase in temperature reduces the production yield of important crops (such as wheat, rice and maize), especially in areas where these crops are already at the limits of their temperature tolerance (mainly in tropical and temperate regions), while floods and droughts contribute to the removal of fertile topsoil [48]. In fact, a number of studies predict a reduction in crop production of up to 50% by 2050 in developing countries in South America, Africa and South/Southeast Asia as a result of a temperature rise by 3 °C [49]. It is estimated that extreme climate conditions could force an excess of 100 million more people into hunger and poverty by 2030 and displace 1 billion people by 2050 [49].

Food availability (referring to absolute food quantities, nutrient quality or dietary diversity) is further reduced by the need for increasing amounts of water for crops in areas affected by drought and increased temperature, a demand often unmet in countries with weak economies. 

Climate change and extreme climatic events also largely impact the livestock and dairy industry [50]. Temperature increase (coupled with other climate phenomena, e.g., floods) impacts the quantity, availability and quality of food and water intended for animals, posing significant challenges in maintaining livestock numbers and managing grazing intensity (especially in pastoralist herder settings) [51]. 

Higher temperatures may also lead to the deterioration of animals’ health and development, affecting food intake levels, animal behavior and their metabolism [52]. Thermal stress is also often accompanied by reduced productivity and fertility and increased vulnerability to infectious diseases [53].

Climate variability and extremes and their underlying ties to food insecurity largely impact the lives of households with the lowest incomes [1]. Food utilization in developing countries and low-income households is undermined by temperature increases and rainfall pattern changes, which often drive and alter the population dynamics of insects, weeds and micro-organisms with detrimental effects on the quantity, quality and safety of stored food [54]. Extreme climate events also affect food distribution via the destruction of roads and other infrastructure, preventing markets from being stocked adequately and in a timely fashion [4].

Moreover, food access for low-income persons is affected by: (a) food price increases and volatility as a downstream effect of climate variability induced food loss at the production/distribution stage, as well as through (b) climate extreme induced diminished employment opportunities in the food (or other) sector, collectively resulting in income loss and inability to reach household food demands [55]. Furthermore, extreme climate events and subsequent environmental degradation reduce developing countries’ and low-income communities’ resilience and adaptive capacity, increasing their vulnerability to food insecurity, malnutrition and food crises [1,56].

#### 3.2.2. Wars/Conflicts

Wars and armed conflicts are a major driver of intense food insecurity. It is indicative that all countries currently facing food crises and/or a high risk of famine are experiencing armed conflicts [57] whilst, according to the World Food Program (WFP), 60% of the world’s hungriest people live in conflict zones [58]. Currently, more than 35 million acutely food insecure people attributed to conflicts are situated in Asia and the Middle East, while conflict driven food insecurity is also reported at high levels in the Lake Chad Basin and Central Sahel (Africa) [4].

Wars and conflicts disrupt food systems and markets in multiple ways. They generate inflation, particularly in food prices (in addition, black markets often flourish in such circumstances) and reduce people’s capacity to produce, trade and buy, collectively resulting in food scarcity [59]. Conflicts also undermine food system infrastructure (e.g., food markets are almost non-existent in Yemen and several regions of Syria) and weaken economies, thus reducing employment and increasing poverty levels, further straining peoples’ access to adequate food [60]. Deterioration in public health systems in conflict zones also has a toll on crop and livestock production due to diseases and sickness [60].

A major side-effect of conflicts is the displacement of large populations, which are acutely susceptible to food insecurity along their treacherous journeys to seek safety and better lives, or upon their obligatory enclosure in refugee camps under poor living conditions where food is often scarce and inadequate in meeting their nutritional needs [4,46,61,62].

#### 3.2.3. Economic Crises and Economic Shocks

In 2009 alone, the global financial and economic crisis forced an additional 100 million people into hunger, resulting in a total of 1 billion undernourished people across the globe [63]. In Europe, the 2008–2009 economic crisis increased food insecurity levels in nearly all countries [64]. Indicatively in 2013–2014, nearly 1 million people in the United Kingdom visited food banks (a proxy measure of food insecurity) [65,66]. Moreover, a study investigating the effect of the economic crisis on food insecurity in Greece found an increasing food insecurity trend over the period 2008–2015, coinciding with the implementation of austerity measures [67,68].

Nonetheless, the global financial crisis of 2008–2009 impacted food security in the developing world, with countries such as Venezuela, Zimbabwe, Haiti and Sudan affected most [4].

Financial and economic crises negatively affect food insecurity at the country and individual level through a number of channels. Economic crises, inherently tied to inflation and currency devaluation, significantly reduce incomes, while food prices stand high, reducing the purchasing power and access of low-income persons and households to adequate and high-quality food [4,69].

Specifically, households with low income spend less on food and tend toward low-cost meals that provide high amounts of sugar, saturated and trans fats, and sodium [70]. Moreover, many foods that are considered healthy (fruits, vegetables, fish) have a high cost, making them less affordable [71]. For instance, healthy dietary choices during austerity in Greece have been influenced negatively by diminishing consumer level financial affordability [72].

Economic crises also increase unemployment rates, hence reducing food purchasing power, while crisis-related factors, such as poor working conditions (e.g., non-conventional working hours, part-time work, etc.), increase food insecurity risk [73].

#### 3.2.4. COVID-19 Pandemic Effect on Food Insecurity

It is difficult to definitively assess and evaluate the pandemic’s full impact on food insecurity levels. Even so, several public health agencies and organizations (e.g., WHO, FAO) estimated in 2021 an increase of at least 83 million undernourished people worldwide, possibly extending to 132 million, predominantly as a downstream result of the SARS-CoV-2 triggered economic crisis [47]. Moreover, in 2020 (the year of strict lockdowns in many countries), European food banks redistributed 68% more food compared to 2019 with a number of countries, including Greece, doubling their food distribution [74].

The COVID-19 pandemic negatively impacted food security levels in both developed and developing countries [66,67,68,69,70,71,72,73,74,75,76]. For example, Nigeria reported significantly increased household food insecurity levels throughout 2020 compared to the pre-pandemic years [75], while 10% of American adults reported a threefold increase in food insecurity compared to pre-pandemic years [76]. Further evidence frοm the USA shows food insecurity prevalence exceeding 30% during the first 4 months of the pandemic in 2020 [77], whilst several USA surveys indicate a steep rise in food insecurity (post-pandemic onset) in households with children, in comparison to previous years [78,79].

The COVID-19 pandemic worsened food insecurity due to a number of reasons. Firstly, during the pandemic’s acute phase, agriculture and food systems were largely crippled (especially in developing countries), thus halting and even reverting economic growth [47]. Lockdown measures affected all stages of the food supply chain, including logistics [80]. Shortages, production falloffs and food export reductions were recorded in many countries, with the International Food Policy Research Institute estimating in 2021 a 25% reduction in agricultural food exports in developing countries [47].

Concomitantly to reduced food production, food prices increased, jointly resulting in reduced food availability and access [47]. Notably, a large number of small-scale farmers, vendors and food distributors utilizing unofficial marketplaces were impacted by the COVID-19 restriction measures, hindering supply delivery [81]. At the same time, food costs skyrocketed making food unaffordable for millions of people living in poverty [81].

Furthermore, the pandemic and associated economic recession led to an overall increase in poverty, unemployment, under-employment and working poverty with devastating downstream effects on food security especially in economically insecure households [3].

The main findings regarding key drivers of food insecurity are summarized in Table 4.

## 4. Discussion

Investigating the magnitude of food insecurity worldwide, we observed high levels of moderate and severe food insecurity across the globe, affecting hundreds of millions of people, impacting most socioeconomically deprived populations and areas. In Greece specifically, we found moderate or severe food insecurity prevalence rates ranging between 6.6% and 8% for the years 2019–2022 [37], necessitating food security action with a prime focus on the most vulnerable populations (i.e., children, refugees and socioeconomically deprived individuals). Furthermore, we identified several interrelated key drivers fueling food insecurity across the globe, including war, climate change and economic crises, with their negative impacts further amplified by the COVID-19 pandemic.

Our review findings align with food insecurity studies and reports available for 2024. According to the WFP, over 309 million people across 72 countries are facing acute levels of food insecurity in 2024 [83]. Specifically, latest evidence from the Gaza Strip describes one of the worst war-driven food crises ever recorded with over two million people currently facing highly acute food insecurity [84]. Moreover, reports from Yemen covering the period October 2023—February 2024 describe over 4.5 million people experiencing high level acute food insecurity, attributed to economic crisis, armed conflict, and climatic extremes [85,86]. Last but not least, food insecurity appears to be worsening in West and Central Africa where 49.5 million people are expected to experience hunger between June and August 2024, corresponding to a 4% increase compared to 2023 [87].

Overall, the world stands far from progressing towards the sustainable development goal 2 “Zero Hunger” and specifically the targets 2.1 “End hunger and ensure nutritious and sufficient food to all”, and 2.2 “Eradicate all forms of malnutrition”, by 2030 [17,88]. More so, amidst the unravelling global multi-crisis, we are standing at a critical crossroads: (a) further increase in food insecurity or (b) the application of new knowledge, tools, competences and good health, as well as inequality reduction policies tackling food insecurity, with an end goal of zero hunger across the globe.

Public health policies, such as intensifying humanitarian aid, including food aid programs [56,89] and setting up food banks, are definitely important relief measures to a number of people experiencing food insecurity [90,91]. However, in the best-case scenario, these measures may improve people’s food insecurity status only on a temporary basis; thus, they do not challenge the underlying causes and mechanisms of food insecurity.

Rather, eliminating food insecurity requires acting upon the interrelations of several sustainable development goals with food insecurity, including “Zero Poverty”, “Climate Action”, “Decent Work and Economic Growth”, “Reduced Inequalities” and “Responsible Consumption and Production” [15,92].

For instance, food insecurity is worsening concomitantly with food overproduction and massive food loss and waste [93,94,95,96], both governed by the existing system’s tendency to provide a constant oversupply of food to the western markets in support of the overconsumption model in place. Indicatively in the EU, approximately 131 kg of food waste per person were generated in 2021 [97].

Reducing food loss and waste, apart from being an ethical priority [98], may enhance food security through improving nutritional status and health in an environmentally sustainable manner [99], and reducing greenhouse gas emissions, a well-known driver of climate change [93,100]. Utilizing, in this direction, cutting edge technology across the food supply chain (including advancements in artificial intelligence, nanotechnology, robotics and automation [101]) may significantly reduce food loss [102].

FAO suggests integrating the right to adequate food in national food and nutrition security policies and programs. Key points of such policies include (a) identifying vulnerable groups, (b) addressing the underlying causes of food insecurity for each group, (c) establishing specific time-bound goals, and (d) integrating food insecurity policies in multisectoral policies [103,104]. Considering the burden of food insecurity in socioeconomically deprived populations, as well as their vulnerability to food insecurity amidst economic crisis contexts, implementing socioeconomic policies improving the financial and employment circumstances of low-income households may significantly decrease food insecurity prevalence and severity [105]. EU policies concerning food security and food crises appear to be receiving greater attention nowadays compared to the past. Between the Seventh Framework Programme (2007–2013) and Horizon 2020 (2014–2020), the number of EU-funded Research and Innovation projects related to food security has more than doubled, increasing from around 200 to over 450. Some projects have already demonstrated successful application, such as *InnovAfrica*, which introduced technologies and approaches to enhance food security in sub-Saharan Africa, resulting in positive impacts on agricultural systems. Additionally, the *DiverIMPACTS* project aimed to fully leverage the diversification of cropping systems, offering various benefits, such as improved food security, and a steady supply of agricultural products for feed [106].

Coupling such efforts with capacity building in developing countries and regional/global food distribution regulation policies aiming to ensure consistent food availability and accessibility in crisis (e.g., war-ridden) settings are essential if we are to curb food insecurity and prevent food crises [107].

Climate change is a key food insecurity driver, posing as essential both system sustainability and resilience against climatic extremes [108]. In a broader sense, the concept of Planetary Health, recently gaining increasing recognition, appears highly promising within the context of addressing climate change and its downstream effects on food insecurity.

Planetary Health focuses on two main interrelated axes: (a) the interactions between human activities and the natural environment and their imprint on the state of environmental and human health; and (b) the sustainability of human civilization, that is, the ability of global society to act in a way that is beneficial to its long-term survival through the challenging of existing political and socio-economic frameworks of human activity [109]. From a strategic standpoint, Planetary Health sets a theoretical framework against the status quo condition of health inequalities and food insecurity for the most vulnerable, through which the 2030 sustainable development goals “Climate Action” and the interrelated “Zero Hunger” may be realized.

Our study has several limitations. Although we thoroughly searched the international literature measuring the magnitude of food insecurity, we do not include here the entirety of available studies potentially meeting our inclusion/exclusion criteria. Hence, certain inter-regional or between-country food insecurity prevalence variations may not be described. A second limitation is that the included studies display a wide range of study designs, including usage of different indicators measuring the magnitude of food insecurity, making strict geographical and temporal comparisons difficult. Furthermore, in the absence of golden standard cut-offs determining the levels of food insecurity (e.g., moderate or severe), interpretation of food insecurity estimates described here by severity level requires caution [110]. Finally, we highlight several major macro-factors driving food insecurity, though other secondary contextual factors acting in conjunction with those identified and described may exist.

## 5. Conclusions

Food insecurity comprises a major global public health threat and currently the world stands far from progressing towards the 2030 sustainable development goal “Zero Hunger”. Key underlying food insecurity macro-drivers include climate change, economic crises and conflicts/wars, whilst the COVID-19 pandemic has further exacerbated food security inequalities to the disadvantage of socioeconomically deprived populations. Tackling food insecurity through addressing its key drivers is essential if we aim to progress towards succeeding in the goal of eliminating hunger worldwide.

## Figures and Tables

**Table 1:** Selected studies evaluating the magnitude of food insecurity (FI) globally.

Study	Population	Methodology	Aim	Indicators of FI	Main Results *
FAO et al. (2021) [1]	Across the globe	Review	To present the first global assessment of food insecurity and malnutrition for 2020	N.A.	Economic downturns as a consequence of COVID-19 containment measures all over the world have contributed to one of the largest increases in world hunger in decades, which has affected almost all low- and middle-income countries and can reverse gains made in nutrition. Between 720 million and 811 million people faced hunger in 2020.
FAO et al. (2023) [25]	Across the globe	Review	To update the global assessment of food security and nutrition and their main drivers	Prevalence of undernourishment (Sustainable Development Goal (SDG) Indicator 2.1.1)	Global hunger remained relatively unchanged from 2021 to 2022 but is still far above pre-COVID-19-pandemic levels. Approximately 2.4 billion people were moderately or severely food insecure in 2022.
Pereira et al. (2017) [26]	Nationally-representative samples from the Gallup World Poll survey (2014–2015)	Observational study	To present the first global and regional estimates of the share and number of children below age 15, who live with a respondent who is food insecure	Food Insecurity Experience Scale (FIES) for the last 12 months	Among 147 countries and four territories, 41% of children under age 15 live with a respondent who is moderately or severely food insecure, 19% live with a respondent who is severely food insecure, and 45% live with a respondent who reported not having enough money to buy food in the previous 12 months
Broussand (2019) [27]	132,983 adults from the Gallup World Poll survey (2014–2015)	Observational study	To investigate the food security in women	Food Insecurity Experience Scale (FIES) for the last 12 months	Women have a higher probability of being food insecure relative to men. The magnitude of the gender gap in food insecurity varies across regions and varies by the severity level of food insecurity
UNICEF (2019) [28]	Children	Report	To assess and describe the magnitude and the underlying drivers of malnutrition globally.	N.A.	In 2019, approximately 149 million children below the age of 5 years were stunted, nearly 50 million wasted or subject to acute undernutrition, 340 million experienced systematic micronutrient deficiencies and 38.2 million were overweight or obese

N.A: Not applicable/Not available. * main study results in respect to the magnitude of food insecurity.

**Table 2:** Selected studies evaluating the magnitude of food insecurity (FI) in Europe.

Study	Population	Methodology	Aim	Indicators of FI	Main Results *
Grimaccia & Naccarato (2022) [2]	Nationally representative samples of European participants in the Gallup World Pool data	Observational study	To investigate the role of gender as a determinant of FI in Europe	Food Insecurity Experience Scale (FIES) for the last 12 months	Variation exists in food insecurity both across EU countries and over time between 2009 and 2012, peaking at >25% in Hungary in 2012.
Tsapogias (2023) [29]	Ν.A	Narrative review	To investigate the impact of climate crisis on food insecurity in Europe	N.A	Europe is characterized by good levels of food security, compared to most continents. However, there are important issues concerning the current and future food security of Europe’s populations, while at the same time the various risks are not equally distributed in the different regions, on a global scale, but also within the European continent
Loopstra (2020) [30]	Populations living in European countries	Observational study	To provide an overview of food insecurity in Europe and what works and what does not work to tackle food insecurity	Food Insecurity Experience Scale (FIES)	Recent data generated via the FAO’s FIES shows that the prevalence rates of food insecurity in European countries range from 3.1% to over 20%.

N.A: Not applicable/Not available. * main study results in respect to the magnitude of food insecurity.

**Table 3:** Selected studies evaluating the magnitude of food insecurity (FI) in Greece.

Study	Population	Methodology	Aim	Indicators of FI	Main Results *
Foundation for Economic and Industrial Research (2017) [35]	Ν.A	Review	Highlight the role of foodbanks for addressing food insecurity and recourse waste.	Ν.A	In 2015, more than 1.4 million people in Greece experienced food insecurity corresponding to 12.9% of the country’s population.
Hellenic Statistical Authority (2023) [37]	10,202 households and 22,317 members of those households participating in the Income and Living Conditions Survey 2022	Observational study	To investigate the level of FI in Greece in 2021	Food Insecurity Experience Scale (FIES)	6.6% of the total population experienced moderate or severe levels of food insecurity, whereas 1.5% of the total population experienced only severe levels of food insecurity
Hellenic Statistical Authority (2021) [38]	15.086 households and 32.962 household members participating in the Income and Living Conditions Survey 2020	Observational study	To investigate the level of FI in Greece in 2019	Food Insecurity Experience Scale (FIES)	6.1% of the population reported experiencing moderate or severe food insecurity;1.6% experienced severe food insecurity
Grammatikopoulou et al. (2019) [39]	207 community-dwelling older adult participants living in Thessaloniki and Kavala, Greece	Cross-sectional study	To assess the prevalence of malnutrition and food insecurity among community-dwelling older adults, and to identify possible factors associated with them	Household Food Insecurity Access Scale (HFIAS)	A large proportion of Greek older adult population is at risk of malnutrition and experience some degree of food insecurity.
Gkiouras et al. (2020) [40]	121 older adults (mean age: 72.6 (± 8.1) years) visiting a Primary Care Health Center at Nea Kallikratia, Northern Greece	Cross-sectional study	to assess the prevalence of food insecurity among older adults and evaluate the association between food insecurity, malnutrition, chronic disease, multimorbidity and healthcare utilization	Household Food Insecurity Access Scale (HFIAS)	An alarmingly high proportion of older adults attending outpatient clinics experience food insecurity, often coinciding with malnutrition.
Theodoridis et al. (2018) [42]	A non-probability sample of 236 students from Athens and Thessaloniki during 2016	Cross-sectional study	To assess Mediterranean diet adherence and FI among university students in Greece.	Household Food Insecurity Access Scale (HFIAS)	The majority of the surveyed university students from Greece demonstrate some degree of FI, with a great proportion (45.3%) being severely food-insecure, 22.0% experiencing moderate FI and the remaining 14.8% with low FI. Increased FI is inversely associated with MD adherence.
Petralias et al. (2016) [43]	162 schools with 25,349 students participated during the 2012–2013 school year	Intervention	To establish the extent of food insecurity and the potential impact of a large-scale school-based nutritional program, in low-socioeconomic status districts of Greece, during the current economic crisis	Food Security Survey Module were assessed at baseline and after a 1–8-month intervention period	Children and families residing in low socioeconomic areas of Greece, experience high levels of food insecurity (i.e., 56.3%)
Dalma et al. (2020) [44]	1442 pre–post intervention questionnaire pairs in the multicomponent intervention (MI: each student received a daily healthy meal along with educational actions; 28 schools) group and 986 in the educational intervention (EI: 23 schools) group	Cluster randomized trial. Schools participating in the DIATROFI Program in Greece during the 2014–2015 school year were randomly allocated between a MI and an EI group	To examine the impact of a school feeding program combining healthy meals provision and educational activities to reduce food insecurity	Food Security Survey Module (FSSM) containing 18 questions	45% of children’s households residing in low socioeconomic areas of Greece experienced high levels of food insecurity.

N.A: Not applicable/Not available. * main study results in respect to the magnitude of food insecurity.

**Table 4:** Selected studies investigating the major macro-factors driving food insecurity (FI).

Study	Population	Methodology	Aim	Indicators of FI	Main Results *
**Climate Change**					
Parry et al. (2005) [48]	Around the globe	Review Report	To describe the outcomes of research assessing the implications of climate change on food production, food supply systems and prevalence of hunger.	Models evaluating the potential alterations in crop yields and the assessment of global responses in food trade.	The prevalence of hunger seems to increase due to climate change, especially in Southern Asia and Africa. In certain regions, this geographical distribution appears to be influenced more by the increase in poverty than by the regional pattern of climate change.
Godde et al. (2021) [50]	Around the globe	Review	To examine the vulnerability of climate-related impact across the land-based livestock food supply chain.	Key factors, including feed and water resources, animal health and production, processing and storage capabilities, labor availability, prices, and overall livestock production.	Although it is evident that climate change will influence the sector across the entire food supply chain, significant uncertainties persist regarding the specific nature, scope, and severity of these impacts.
Godde et al. (2020) [51]	Around the globe	Review	To comprehend the degree to which climate change poses a threat to global rangelands.	A global rangeland model combined with datasets on livestock and socio-economic factors were utilized.	Half of the global rangeland areas are forecast to undergo a simultaneous decrease in mean biomass. The main threats to livestock are not solely tied to climate change patterns but also to climate variability and extreme weather events.
Filipe et al. (2020) [52]	Around the globe	Mini review	To provide an update on the alterations to the immune systems of livestock and other animals resulting from phenomena associated with climate change.	N.A.	Climate change significantly affects the production and reproduction of livestock, resulting in substantial financial setbacks. High-yield animals are particularly vulnerable and are often the most severely affected by environmental alterations. Climate change may also affect the immune systems of animals, resulting in immune suppression and heightened susceptibility to infections.
Sevi and Caroprese (2012) [53]	Around the globe	Critical review	Explore the impact of heat stress on sheep production performance, milk quality, immunological state, and udder health.	N.A.	Despite the fact that sheep are heat tolerant species, exposure to high ambient temperatures has a detrimental impact on their production performance, including milk nutritional and technological properties.
Farooq et al. (2022) [54]	Around the globe	Review	To describe the effects of climate change on food production systems, as well as the societal and economic factors influencing unbiased food distribution.	N.A.	Climate change, food production, and food security are inextricably interconnected. Climate change will likely exacerbate food insecurity challenges in areas which are already susceptible to climate change.
**Wars and armed conflicts**					
Food Security Information Network. 2020 [4]	Around the globe	Report	To evaluate acute food insecurity situations globally, involving 16 international partner organizations.	N.A.	Between 2019 and 2020, the global population experiencing food crisis increased from 112 to 123 million. Insecurity and displacement of populations are severe consequences of war or conflict. Over 35 million people experiencing acute food insecurity due to conflicts reside in Asia and the Middle East. Conflict-driven food insecurity is also prevalent in the Lake Chad Basin and Central Sahel regions of Africa.
Grammatikopoulou et al. (2019) [46]	192 refugee children in reception centers in northern Greece	Pilot cross-sectional study	To evaluate the prevalence of acute and chronic malnutrition.	N.A.	A proportion of 7.8% of the children residing in the camps were classified as underweight, 4.6% as wasted, 7.3% as stunted, and 13.0% were found to have experienced at least one form of malnutrition.
Brück and Errico (2019) [57]	Around the globe	Mini review	To explore the connections between recent developments in food security and violent conflicts.	N.A.	Violent conflicts emerge as a primary factor driving food insecurity through several mechanisms.
Heinrich Böll Stiftung (2021) [59]	Around the globe	Report	To address the social and political causes of hunger and malnutrition.	N.A.	In 2019, conflicts were the trigger for six of the ten worst food crises. All countries that experienced famine in 2020 were affected by violent conflict The ways in which conflicts affect food security and agriculture depend on the local situation.
Human Appeal (2018) [60]	Populations living in Syria and Yemen during the armed conflicts	Report	To fight the effects of hunger and malnutrition throughout Syria and Yemen.	N.A.	Since October 2015, the estimated number of Syrian people unable to obtain the basic food required to meet their needs has risen to 38% of the population. A further 4 million are at risk of becoming what is described as acutely “food insecure”. In Yemen, Food Insecurity has 20% since June 2016, with over 60% of the population—17 million people—being food insecure.
**Economic crises**					
Food Security Information Network Global 2020 [4]	Around the globe	Report	To evaluate acute food insecurity situations globally, involving 16 international partner organizations.	N.A.	Economic crises via inflation and currency devaluation significantly reduce incomes, while food prices stand high, reducing the purchasing power and access of low-income persons and households to adequate and high-quality food. The global financial crisis of 2008–2009 impacted most food security in the developing world, with countries such as Venezuela, Zimbabwe, Haiti and Sudan affected most
F.A.O (2009) [63]	Around the globe	Report	To evaluate food insecurity globally	N.A.	In 2009, the global financial and economic crisis forced an additional 100 million people into hunger, resulting in a total of 1 billion undernourished people across the globe
Mittal (2009) [64]	Around the globe	Report	To examine the 2008 global food price crisis, identifying long- and short-term causes	Ν.A.	In Europe, the 2008–2009 economic crisis increased food insecurity levels in nearly all countries
The Trussell Trust (2015) [65]	Adults and children living in the U.K.	Online data collection system into which foodbanks enter the data from each foodbank voucher	To examine the number of persons visiting food banks in the UK in 2014–2015.	People visiting food banks and receiving three days’ emergency food.	In 2014–2015, nearly 1 million people in the United Kingdom visited food banks (a proxy measure of food insecurity)
Konstantinidis (2022) [68]	Representative sample of households, in Greece	Statistics on Income and Living Conditions dataset (SILC) microdata for 2009 and 2014 for Greece, acquired from Eurostat.	To examine the differential effect of the crisis for food insecurity across different types of households.	Affordability of a meal with meat, chicken or fish (or vegetarian equivalent) every second day	The economic crisis had a serious impact on food insecurity in Greece. An increasing food insecurity trend was found over the period 2008–2015, coinciding with the implementation of austerity measures
Brinkman et al. (2010) [69]	Around the globe	Research article	To evaluate the effects of crises on food consumption, nutrition, and health.	Risk analysis using the cost of the food basket, assessment surveys	The global economic crisis increased the cost of the food basket across several countries, forcing households to reduce both the quality and quantity of the food they consumed.
Bonnaccio et al. (2016) [71]	Mediterranean countries	Review	Το provide an overview of the evidence on the current major determinants of adherence to the Mediterranean diet, with a particular emphasis on Mediterranean Countries at a time of economic crisis.	N.A.	Many foods that are considered healthy have a high cost, making them less affordable for low-income households.
Koulierakis et al. (2022) [72]	A representative sample of 2003 individuals living in Greece	Cross-sectional 2016 “Health and Welfare” study.	To investigate the key determinants of dietary choices of the Greek population during a period of financial austerity.	Dietary behaviour was assessed through a set of four questions.	Healthy dietary choices during austerity in Greece have been influenced by financial affordability, unemployment, as well as other demographic characteristics. Fiscal measures and inflation increased food insecurity, and consumers have been affected by the general financial environment.
Huang et al. (2016) [73]	27.497 adolescents form the Survey of the Income and Program Participation, SIPP (2008–2011).	Longitudinal survey conducted at four-month intervals.	To access the correlation between unemployment and household food insecurity amidst the 2007–2009 economic crisis in the USA.	Five questions extracted from an eighteen-item food security scale.	For households facing equivalent durations of unemployment, each additional occurrence of unemployment increased the probability of experiencing food insecurity by 8%.
**COVID-19 pandemic**					
Kapodistrias et al. (2022) [74]	N.A.	Multiple case study	To analyze the effects of the COVID-19 crisis on the operations of European food banks and assess their resilience in managing the pandemic in 2020.	N.A.	Food banks in 2020 managed to distribute a considerably larger quantity of food despite facing numerous social restrictions and other challenges brought on by the pandemic. The implementation of novel strategies and internal organizational structures, alongside the establishment of new types of external network relations with other companies and/or public organizations, proved to be especially crucial.
Avery (2021) [80]	N.A.	Commentary	To describe the evidence suggesting a relationship between food insecurity, malnutrition, and alterations in intestinal microbial composition.	N.A.	COVID-19 resulted in an increase in global food insecurity through several mechanisms (e.g., through enhancing unemployment/under-employment and affecting all stages of the food supply chain).
Gebeyehu et al. (2023) [82]	N.A.	Systematic Review	To provide a summary of the food security impact of COVID-19, highlighting the dimensions of food security that have been critically affected.	N.A.	Ten studies confirmed that COVID-19 had significantly impacted food security, affecting individuals, households, and the broader community, leading to food insecurity. Among the included studies, nine argued that the dimension of food accessibility was the most compromised.
Martinez (2021) [75]	Data from the Nigerian General Household Survey 2018/19 and the first seven rounds of the LSMS-ISA National Longitudinal Panel Survey on COVID-19 collected between April and November 2020.	Longitudinal study	Τo examine trends in household food insecurity in Nigeria just after lockdowns were imposed and at multiple points later in 2020, and to assess the impacts of lockdowns in spring 2020 on household food insecurity.	Food Insecurity Experience Scale (FIES), before and during the COVID-19 pandemic	Household food insecurity in Nigeria notably increased between January and February 2019 and the early months of the COVID-19 pandemic in April/May 2020.
Census Bureau’s Household Pulse Survey (2021) [76]	Adults living in the US.	Research study	To deploy quickly and efficiently, collecting data on critical social and economic matters affecting U.S. households	N.A.	A proportion of 10% of American adults reported a threefold increase in food insecurity compared to pre-pandemic years

N.A. Not applicable/not available. * main study results in respect to the magnitude of food insecurity.

## Data Availability

The original contributions presented in the study are included in the article, further inquiries can be directed to the corresponding author.
